# Mathematical modeling of intraplaque neovascularization and hemorrhage in a carotid atherosclerotic plaque

**DOI:** 10.1186/s12938-021-00878-4

**Published:** 2021-04-29

**Authors:** Yan Cai, Jichao Pan, Zhiyong Li

**Affiliations:** 1 School of Biological Sciences & Medical Engineering, Southeast University, Nanjing, China; 2 School of Mechanical, Medical and Process Engineering, Queensland University of Technology, Brisbane, QLD 4001 Australia

**Keywords:** Mathematical modeling of vulnerable plaque, Intraplaque neovascularization and hemorrhage, Dynamics of plaque microenvironmental factors

## Abstract

**Background:**

Growing experimental evidence has identified neovascularization from the adventitial vasa vasorum and induced intraplaque hemorrhage (IPH) as critical indicators during the development of vulnerable atherosclerotic plaques. In this study, we propose a mathematical model incorporating intraplaque angiogenesis and hemodynamic calculation of the microcirculation, to obtain the quantitative evaluation of the influences of intraplaque neovascularization and hemorrhage on vulnerable plaque development. A two-dimensional nine-point model of angiogenic microvasculature is generated based on the histology of a patient’s carotid plaque. The intraplaque angiogenesis model includes three key cells (endothelial cells, smooth muscle cells, and macrophages) and three key chemical factors (vascular endothelial growth factors, extracellular matrix, and matrix metalloproteinase), which densities and concentrations are described by a series of reaction–diffusion equations. The hemodynamic calculation by coupling the intravascular blood flow, the extravascular plasma flow, and the transvascular transport is carried out on the generated angiogenic microvessel network. We then define the IPH area by using the plasma concentration in the interstitial tissue, as well as the extravascular transport across the capillary wall.

**Results:**

The simulational results reproduce a series of pathophysiological phenomena during the atherosclerotic plaque progression. It is found that the high microvessel density region at the shoulder areas and the extravascular flow across the leaky wall of the neovasculature contribute to the IPH observed widely in vulnerable plaques. The simulational results are validated by both the in vivo MR imaging data and in vitro experimental observations and show significant consistency in quantity ground. Moreover, the sensitivity analysis of model parameters reveals that the IPH area and extent can be reduced significantly by decreasing the MVD and the wall permeability of the neovasculature.

**Conclusions:**

The current quantitative model could help us to better understand the roles of microvascular and intraplaque hemorrhage during the carotid plaque progression.

## Introduction

Atherosclerosis is the process in which plaques, consisting of lipoproteins, monocyte/macrophages, vascular smooth muscle cells (SMCs) and platelets, are built up in the injured walls of arteries as a chronic inflammatory response. In the early phase of the plaque development, hypercholesterolemia conditions increase low-density lipoprotein (LDL) infiltration and retention into the injured endothelial layer in response to disturbed blood flow pattern, leading to the accumulation of inflammatory cells by the release of pro-inflammatory factors, such as monocyte chemoattractant protein (MCP-1). Meanwhile, vascular SMCs undergo phenotypic dedifferentiation that from quiescent phenotype to a synthetic and activated phenotype, in response to pro-inflammatory cytokines and oxidized LDL (ox-LDL). As the atherosclerotic plaque develops, a hypoxic microenvironment in the plaque lesion is forming due to the increased oxygen consumption caused by high metabolic active cells (such as macrophages). This pathological microenvironment will trigger intraplaque neovascularization by upregulation of pro-angiogenic factors, such as vascular endothelial growth factors (VEGFs). The angiogenic microvessels generated from the adventitial vasa vasorum transport blood-borne components including inflammatory cells and lipoproteins into the lesion, which can exacerbate the lipid and inflammatory microenvironment inside the plaques [1, 2]. Growing evidence has demonstrated the pathological morphological and functional properties of the neoangiogenic vessels, such as the disorganized morphology, the loss of basement membrane and pericytes, and the abnormal wall permeability of the microvessels [3–5]. The leaky and unstable intraplaque microvessels serve as a source of intraplaque hemorrhage (IPH) which influences the plaques to develop vulnerable lesions [6, 7]. A remarkable relationship between IPH and subsequent clinical events, including accumulation of lipoproteins, exacerbated inflammation, disintegrated extracellular matrix (ECM), NC expansion, and thinning of the fibrous cap (FC), has been well established in recent decades [8].

To analyze the interactions between IPH and other pathophysiological phenomena during atherosclerotic plaque progression, animal models have been developed. In these in vitro experiments, IPH can be detected by histological staining. In clinically, high-resolution MR imaging has been used to detect vulnerable plaque properties such as IPH. However, nowadays the in vivo high-resolution imaging technique cannot reveal the microvasculature and the hemodynamics of the microcirculation inside the atherosclerotic plaque. Therefore, the quantitative analysis of intraplaque neovasculature and hemorrhage is unavailable by using clinical imaging data. Fluid dynamic theoretical and computational models have been successfully developed and validated for hemodynamics simulation on carotid artery based on patient-specific imaging data [9–11]. In parallel, the simulations on blood flow and mass transport through microvascular networks have broadened our understanding of the complex mechanisms involved in the microcirculation [12–16]. Based on the two-phase continuum model proposed by Pries et al. [17], mathematical models have been able to take account of large heterogeneities in vascular morphology (i.e., lengths and diameters of microvessels, microvessel density, permeability of microvessel wall) as well as in hemodynamic variables (i.e., hematocrit, blood velocity), which constitute the fundamental characteristics of microcirculation, including intracortical microcirculation [18–20], blood perfusion and oxygen transport in capillaries of skeletal muscle [21] and hemodynamics in angiogenic microvessels of solid tumors [22–24].

In this paper, we perform a series of simulations of angiogenesis in atherosclerotic plaques and hemodynamics in the intraplaque microcirculation. The simulation region and the initial condition settings are based on a histology image of a patient’s carotid plaque. The neovasculature is numerically generated based on the angiogenesis modeling developed in our previous work [22]. We assume the neovasculature is generated by the migration and proliferation of endothelial cells (ECs), which are determined by the chemotaxis movement in response to the VEGF gradient and the adherence with ECM. In addition, we perform the hemodynamic calculation by coupling the intravascular blood flow with the transvascular plasma flow and the interstitial fluid flow throughout the surrounding tissues. The IPH is assumed to associate with the diffusional and convectional transport of extravascular plasma in the interstitial area. The simulational results reproduce a series of pathophysiological phenomena during the atherosclerotic plaque progression, such as the high microvessel density region at the shoulder areas, the enlarged necrotic core, and the IPH caused by the extravascular plasma flow across the leaky wall of the neovasculature. In addition, the simulation results are verified by the experimental data and clinical MR images. The second aim is to investigate the quantitative effects of certain microenvironmental factors on intraplaque hemorrhage, such as the microvessel density (MVD) and the wall permeability of the neovasculature. It is found that the IPH area and extent can be reduced significantly by decreasing the MVD and the wall permeability of the neovasculature.

## Results

Each time step increment (*T* = *T* + 1) corresponds to 150 simulation steps (approximately 1 month). The simulation of neovascularization ends at *T* = 13, when the growth of neovasculature and angiogenic sprouts are both zero. In the following results, the hemodynamics analysis is examined at six stages of angiogenesis during *T = *1 to *T* = 13.

### Flow in the angiogenic capillary network

**Fig. 1:**
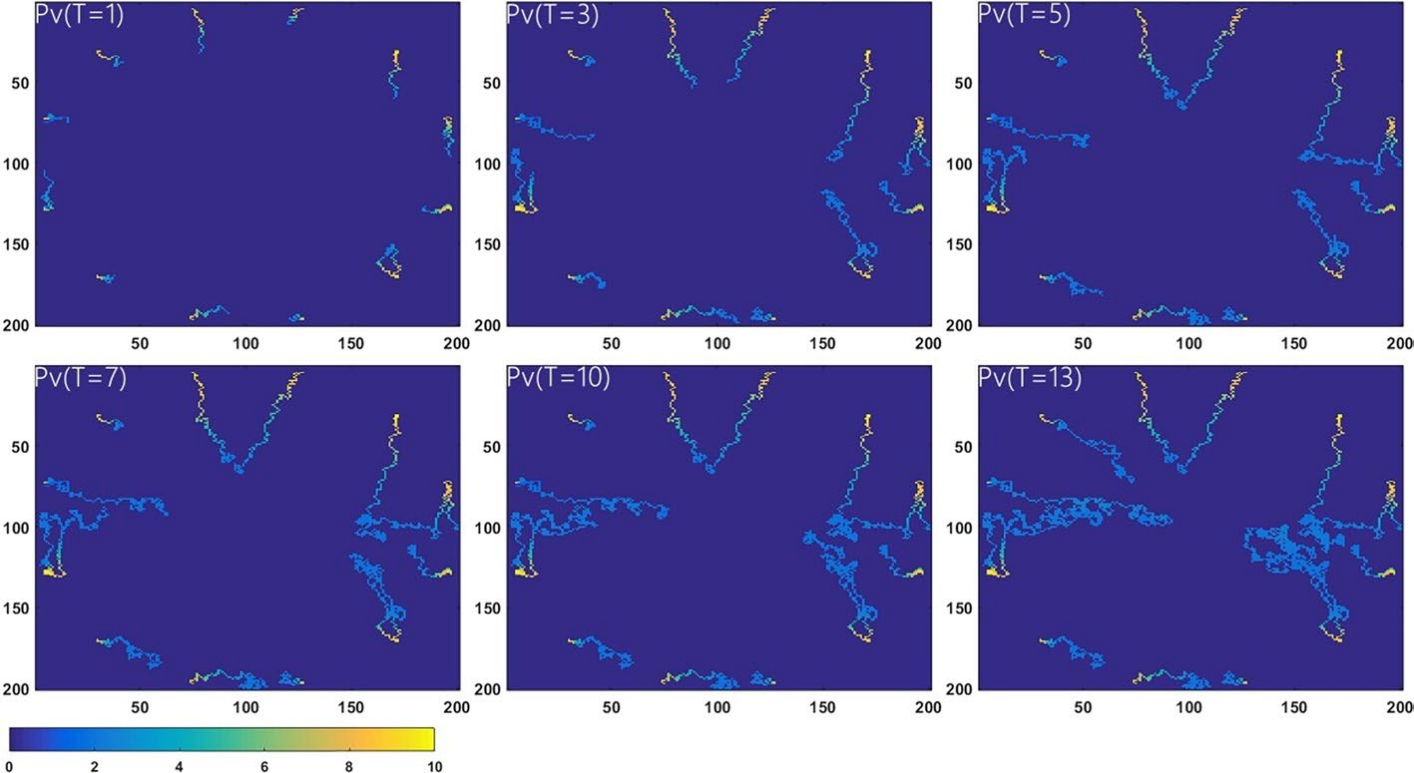
Spatial distribution of the intravascular pressure Pv in the induced capillary network at six different stages

Figure 1 displays the change of $P_{V}$ with structures of the neovessels at six different stages. The neovasculatures are most numerous in the shoulder area, and growing into the core region gradually. As time passing by and with increased neovessels, intravascular pressure $P_{V}$ decreases associate with intravascular velocity $U_{V}$ monotonously from the vasa vasorum of adventitia to the necrotic region. It is found that most reduction of $P_{V}$ and $U_{V}$ occur at the first branching of the new vasculature. The mean values of $P_{V}$ and $U_{V}$ decrease up to 57% through the whole neovasculature.

### Flow in the interstitial space

**Fig. 2:**
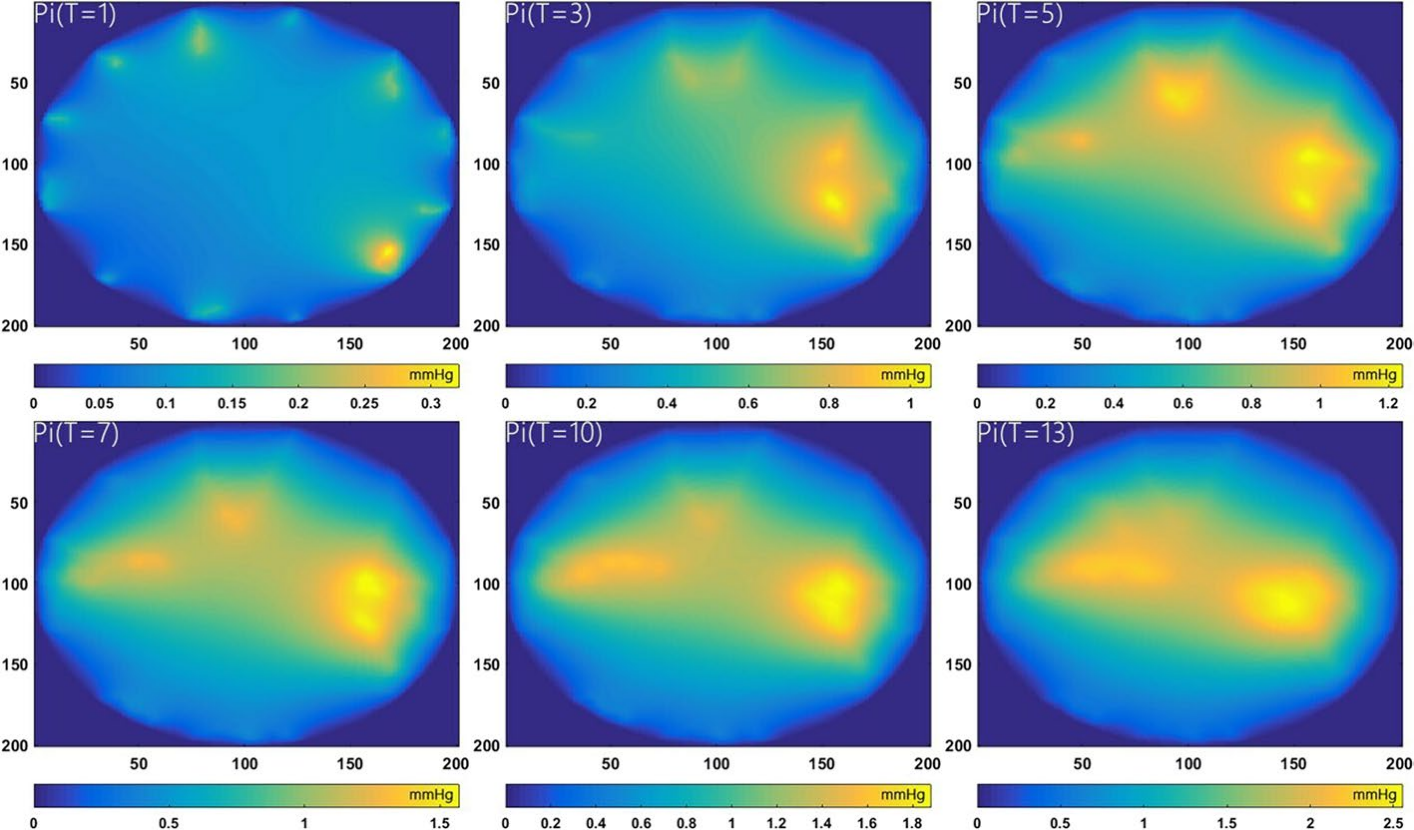
Spatial distribution of the interstitial fluid pressure Pi at six different stages

Figure 2 shows the dynamic changes of the interstitial fluid pressure $P_{i}$ inside the atherosclerotic plaque. The increased $P_{i}$ is observed as early as *T* = 3 at the shoulder area. It is noteworthy that the value of $P_{i}$ and the distribution of high $P_{i}$ both enlarge with the progression of neovascularization (see Fig. 1). At the late stage of plaque development (*T* = 13), the $P_{i}$ has risen to 2.5 mmHg, comparing with the normal interstitial fluid pressure is about zero mmHg. It is noteworthy that the shoulder areas of the plaque are always in a state of interstitial hypertension, which contributes to the intraplaque hemorrhage and plaque vulnerability. This result shows consistency with our previous simulation [25] on the theoretical model.

### Intraplaque hemorrhage

**Fig. 3:**
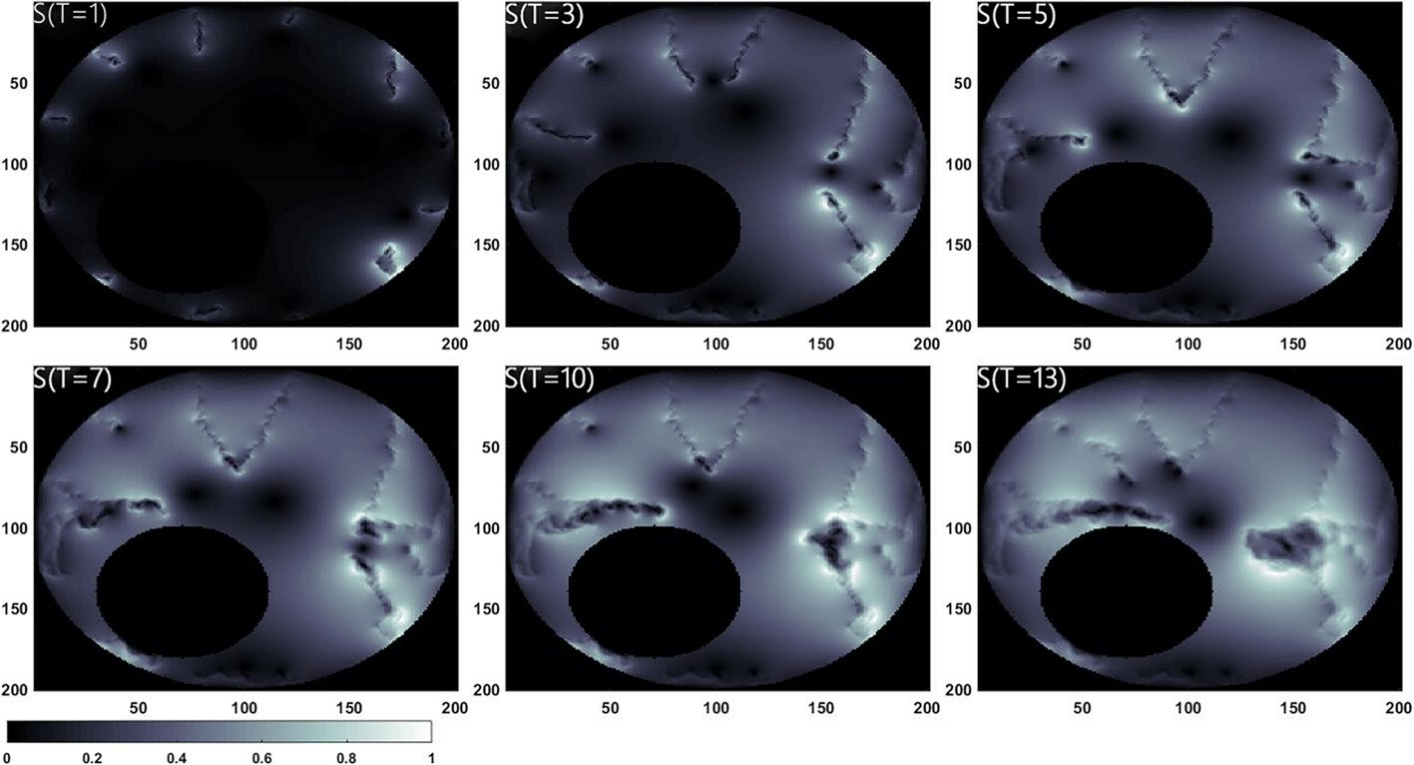
Phenomena of intraplaque hemorrhage at six different stages

The spatial distribution of $S_{IPH}$ is presented in Fig. 3. The highlighted areas denote serious IPH, which are found to be adjacent to the neovasculature. Due to the increasing MVD distributed at the shoulder regions (see Fig. 1), the hemorrhage in the same areas is shown to be higher than that inside the necrotic core especially after *T* = 7. However, the high IPH region gradually spreads from the media to the lipid core.

### Comparison of simulation results with MR imaging

**Fig. 4:**
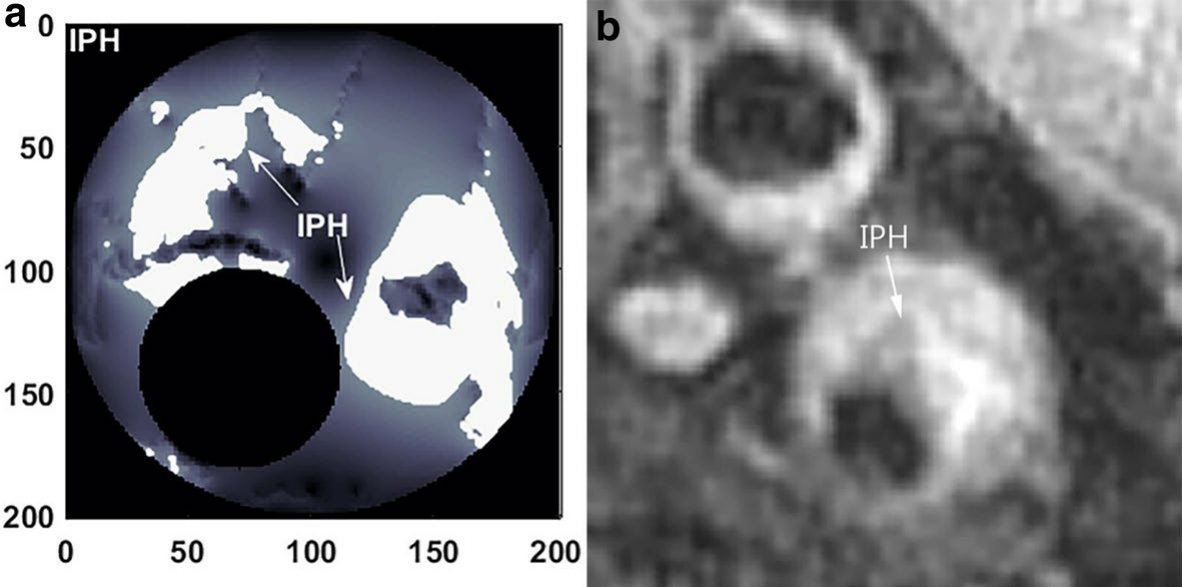
Simulated results of intraplaque hemorrhage (**a**) and MR image of plaque (**b**). The white areas in (**a**), corresponding to the dark areas in (**b**), shows the IPH region mostly distributed in the shoulder areas of the plaque

We set different thresholds ranging from half of the average pixel values to 2.0 times of that value in increments of 0.1 times, to examine the sensitivity of high IPH areas to the segmentation threshold $S_{IPH}^{Thr}$. The optimized threshold $S_{IPH}^{Thr}$ was set to be 1.3 times of the average pixel value to minimize the error with IPH area shown in the MR image. As shown in Fig. 4, it can be found that the white areas in the simulation result, corresponding to the dark areas in the MR image, shows the high IPH region mostly distributed in the shoulder areas of the plaque.

### Comparison of simulation results with in vitro data

**Fig. 5:**
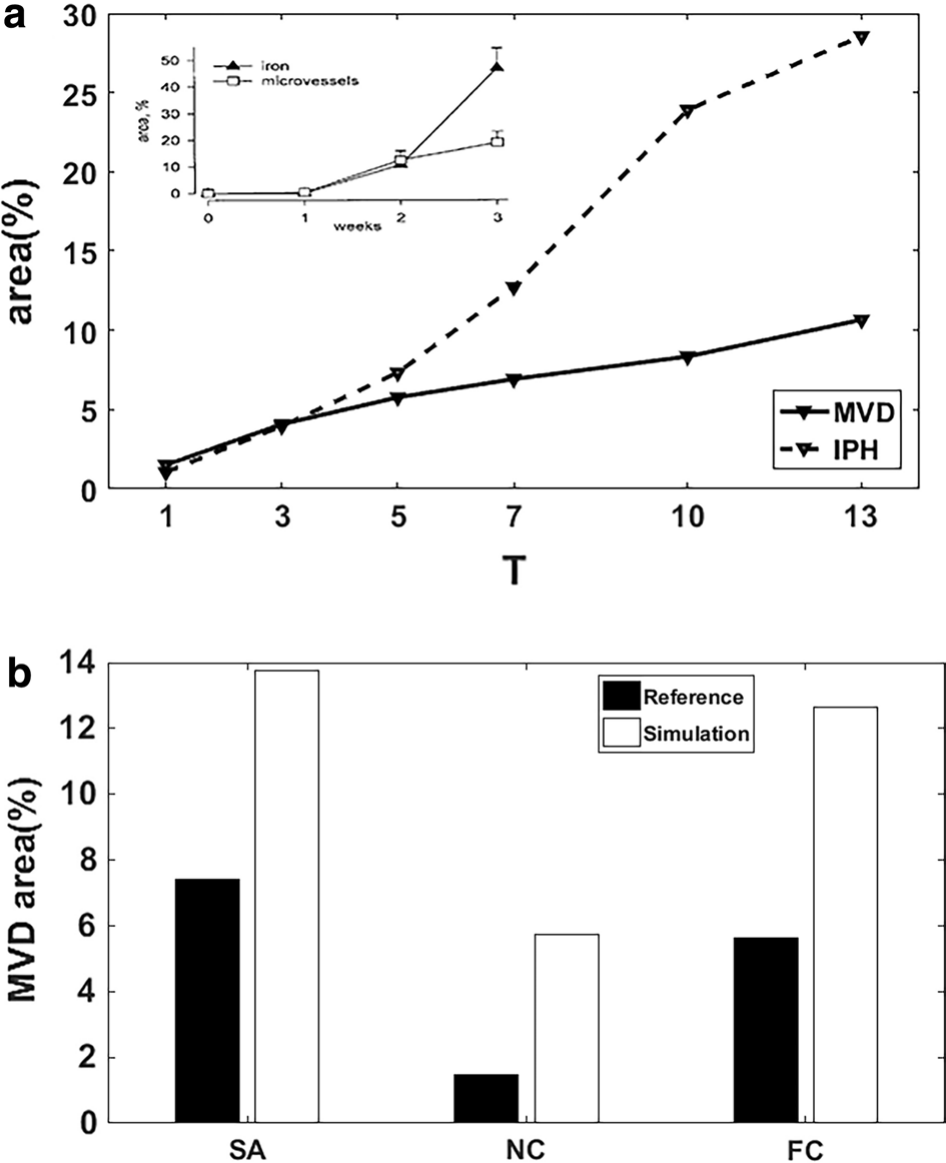
Comparison of simulation results with in vitro data. **a** The growth history curves of MVD and IPH area; **b** MVD in different regions of plaque

The growth history curves of MVD and IPH area are presented in Fig. 5a. The MVD of neovasculature increases continually in the simulation with time, which is consistent with the in vitro histological data in rabbits’ atherosclerotic plaque [26] (see inserted figure in Fig. 5a). The broken line shows a sharp increase of IPH in the late-stage coincided with a significantly elevated iron area observed in the experiment.

Evidence has shown that the MVD varied in different regions of plaque. MVD in the necrotic core is lower than it in the shoulder and cap regions. The simulation results (white bar) show the same characteristic with the experimental observation (black bar) [27] in Fig. 5b. However, the MVD values are a bit higher in simulation results than in the experiment, partly due to the cluster of microvessels decrease the MVD counting in the histological examination.

### Sensitivity analysis of IPH to model parameters

**Fig. 6:**
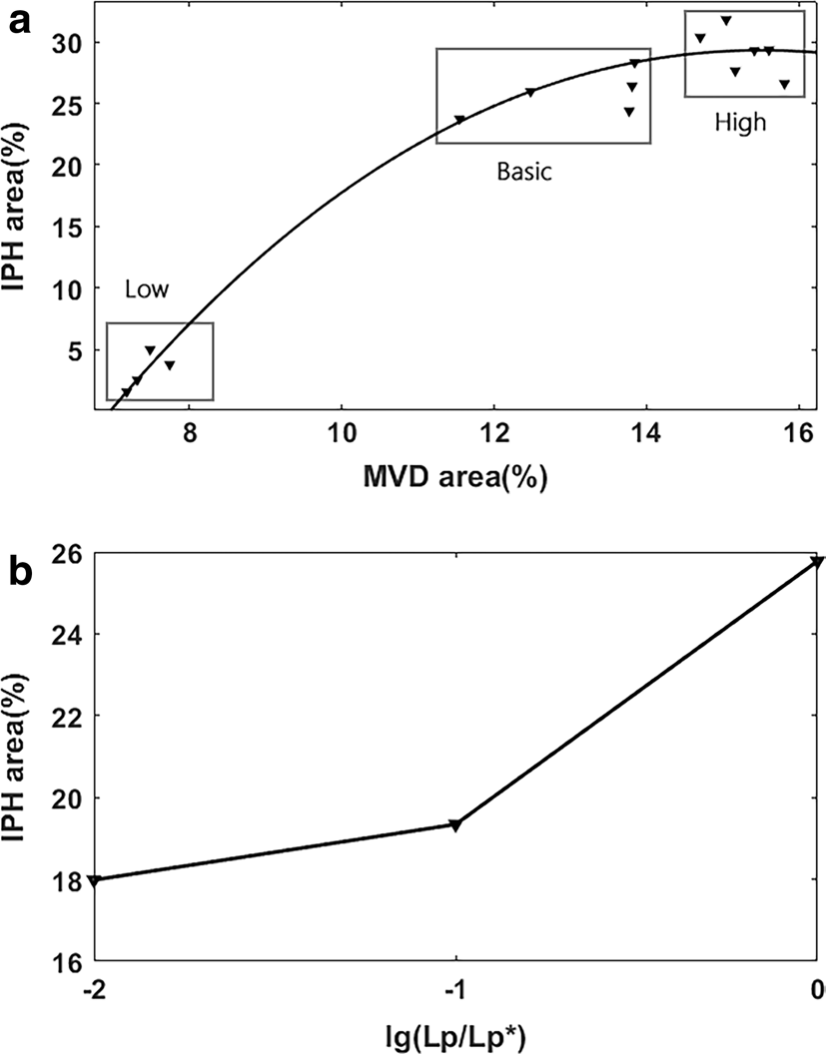
Sensitivity analysis of IPH to model parameters. **a** Investigation of the influence of varied MVD on the IPH area; **b** Influence of the permeability of the angiogenic vessel wall to IPH area

In the present model, the factors that directly influence the IPH involved the MVD and the capillary permeability. To test the system responses to varied MVD conditions, more simulations are performed with increased and decreased MVD. As shown in Fig. 6a, we consider three groups with different MVD (high MVD group, low MVD group, and basic MVD group). The fitting curve shows that the IPH area increases with the MVD of the neovasculature. In addition, the change of IPH area caused by MVD tends to be stable when the MVD value was elevated.

The wall permeability of the intraplaque angiogenic microvessel was assumed to be larger than that in the normal capillary. Here, we reduce the high permeability of capillaries ($L_{P}$). As shown in Fig. 6b, we find that the IPH area decreases almost 30% with a low permeability setting of the microvessels. This result suggests that the abnormal function of intraplaque neovasculature could intensify the pathophysiological microenvironment and the IPH area.

## Discussion

Although the formation and progression of atherosclerotic plaques are understood to be mainly driven by systemic factors, both in vitro and in vivo observations over the past several decades have established that plaque development involves a complex coordination of systemic and local microenvironmental factors that determine how plaques progress. Uncovering the underlying mechanisms and crosstalk of the plaque microenvironment constitutes as a prerequisite for the exploration of possibilities targeting the processes within the plaque microenvironment as novel therapeutic strategies. However, there is currently a paucity of data on the dynamic interactions of the plaque microenvironmental components, due to the unavailability of imaging techniques to quantitatively investigate the changes of plaque microenvironment in both animal experiments and clinical settings. In this context, mathematical modeling provides a potential way to assess the dynamics of the plaque microenvironment by describing the spatial–temporal changes of key cells and chemicals according to physical principles and known pathophysiological interactions. To this end, we performed the evolutional simulations of plaque microenvironmental dynamics. The simulative area was separated into the necrotic core, fibrous cap, shoulder, and media according to the actual layout of plaque histology. The neovasculatures are assumed to sprout and proliferate from the adventitial vasa vasorum, and be attracted and interacted with the local chemicals gradient (such as VEGF, ECM and MMPs). These chemical substances are assumed to distribute unevenly in the simulation region as initial conditions, and transport throughout the whole lesion by diffusion and reaction with each other. 2D9P angiogenesis model are carried out to generate the intraplaque neovasculatures. Simulation results demonstrated the process of intraplaque neovascularization for a period of up to 3 weeks. At the late development stages, the structures of neovasculature have shown typical pathophysiological features observed in the experiments, which include high MVD regions distributed at the shoulder regions as well as a low MVD region at the necrotic core. The hemodynamic calculation of neovaculature is performed by considering the full coupling of the intravascular blood flow with the transvascular plasma flow and the interstitial fluid flow. Based on the simulation results, the extravascular plasma distribution is calculated according to the transvascular plasma flow from the leaky vessel wall and the convective transport within the interstitial tissue, which is used to model the dynamic progression of the IPH area. The hemodynamic results have shown significant consistency with either the histology or the MR images in both quality and quantity ground, including the increased interstitial pressure due to the transvascular plasma flow and the enlarging IPH extent at the shoulder region of the plaque lesion.

The main objective of this work, to investigate the progression of neovascularization and the changes of hemodynamic microenvironment in the atherosclerotic plaques by using a mathematical modeling system, is successfully achieved. Furthermore, we perform the quantitative analysis of the changes of IPH in response to different microenvironmental factors, including the varied MVD and the permeability of the microvessel wall. Simulation results reveal the quantitative relationship between IPH and the varied microenvironmental factors. It is found that the IPH area can be reduced by decreasing the MVD and permeability. Being comprised of different parameters (such as VEGF) that are easily modified, the present model has the potential to incorporate anti-angiogenic progress. Coupled with drug delivery modeling, we envision that our model and its future advances can serve as a theoretical platform for studying the anti-angiogenic therapy applied on atherosclerosis.

IPH is demonstrated to be a major factor in vulnerable plaque development and associated with subsequent clinical events including plaque rupture. However, most of our knowledge about IPH is obtained from the in vitro autopsy studies or animal studies nowadays. This mathematical model would be of great assistance in the set-up of clinical researches by providing a possibility to calculate the IPH dynamics based on patient-specific images. To access the influence of the pre-defined threshold on the comparison of simulation results with MR images, different thresholds are examined to find an appropriate setting for this case. However, more clinical and histological data are needed to validate the functional modules involved in the model.

Nowadays, several imaging modalities are used to characterize one or more plaque microenvironmental factors in vivo. For instance, dynamic contrast-enhanced MRI (DCE-MRI) is proposed to study the intraplaque microvasculature quantitatively and to test the relationship between adventitial perfusion and IPH, while 18F-FDG PET-CT is widely used to evaluate plaque inflammation. Compared with the conventional structural imaging techniques identifying the site and severity of luminal stenosis, these functional assessments may provide more informative values in studying the dynamic microenvironment and consequently evaluate plaque vulnerability. However, it is unrealistic to carry out multi-modality imaging for every patient due to technological and socio-economic issues. In this context, this proof-of-concept study aims to present a quantitative evaluation for atherosclerotic plaque microenvironment by using a generalized mathematical modeling system. The power of mathematical modeling lies in its ability to reveal the underlying dynamic mechanism and the physical principles that might have been overlooked in previous traditional studies. At its best, mathematical modeling provides quantitative supplementary for the imaging data, and enables us to make predictions and early identify which plaque rupture is likely to occur, and leads to a novel and improved ability to assess plaque vulnerability. This will allow actions to be taken in a timely manner to reduce the risk of eventual fatal events on an individual basis. Although we have demonstrated the simulation based on MR images in this study, further researches should be addressed by incorporating other available imaging data to expand the application of this mathematical model in clinical assessment.

With the information of plaque microenvironment as initial input, including plaque morphology and composition obtained by imaging technique and biochemical indicators by blood examination, the dynamics, and development of the main microenvironmental factors can be calculated by using the developed model system. Based on these results, the evolutional progress of a specific plaque can be predicted. The evaluation indicators of plaque microenvironment include lipid deposition, inflammation, IPH, and apoptosis of SMCs. It has been suggested that plaque progression is a modifiable step in the evolution of atherosclerotic plaque. Therefore, a dynamic and quantitative description of the plaque microenvironment can provide direct information for personalized treatment to improve long-term outcomes. We believe the improved evaluation system can help us better understand the interactions among plaque microenvironmental factors and may allow us to predict a possible development of a plaque on an individual basis.

## Study limitations

Despite the successful simulations on the dynamic process of intraplaque angiogenesis and IPH and induced changes in the plaque microenvironment, some pathophysiological facts are simplified in the model set-up. For example, the progress of plaque growth and evolution is not coupled with neovascularization. In fact, the hemorrhage into the necrotic core may increase the core size to some extent and result in luminal narrowing. It will be adapted in the future model by involving the plaque growth in response to the microenvironment caused by the intraplaque neovascularization and IPH. Due to the lack of experimental data, some parameters are estimated in the model, such as the permeability of neovessels. More realistic adjustments will improve the model accuracy. Development of 2D model to 3D model may provide more directions and spaces for intraplaque neovascularization and IPH, which should be addressed in future work.

## Conclusions

A mathematical modelling system was established by incorporating intraplaque angiogenesis and hemodynamic calculation of the microcirculation, to obtain the quantitative evaluation of the influences of intraplaque neovascularization and hemorrhage on the carotid plaque progression. The simulations of the model not only reproduced a series of pathophysiological phenomena during the plaque development, including the high microvessel density region at the shoulder areas, the enlarged necrotic core, and the intraplaque hemorrhage, but also revealed the quantitative relationship between intraplaque hemorrhage and the varied microenvironmental factors. The simulational results demonstrated that the IPH area can be reduced by decreasing the MVD and the permeability of the neovasculature. The current quantitative model could help us to better understand the roles of microvascular and intraplaque hemorrhage during the carotid plaque progression. Additionally, with more patient-specific data, the model could be improved to predict the possible development of an individual plaque based on the dynamic interactions of the plaque microenvironmental factors.

## Methods

### Setup of angiogenic microvasculature

**Fig. 7:**
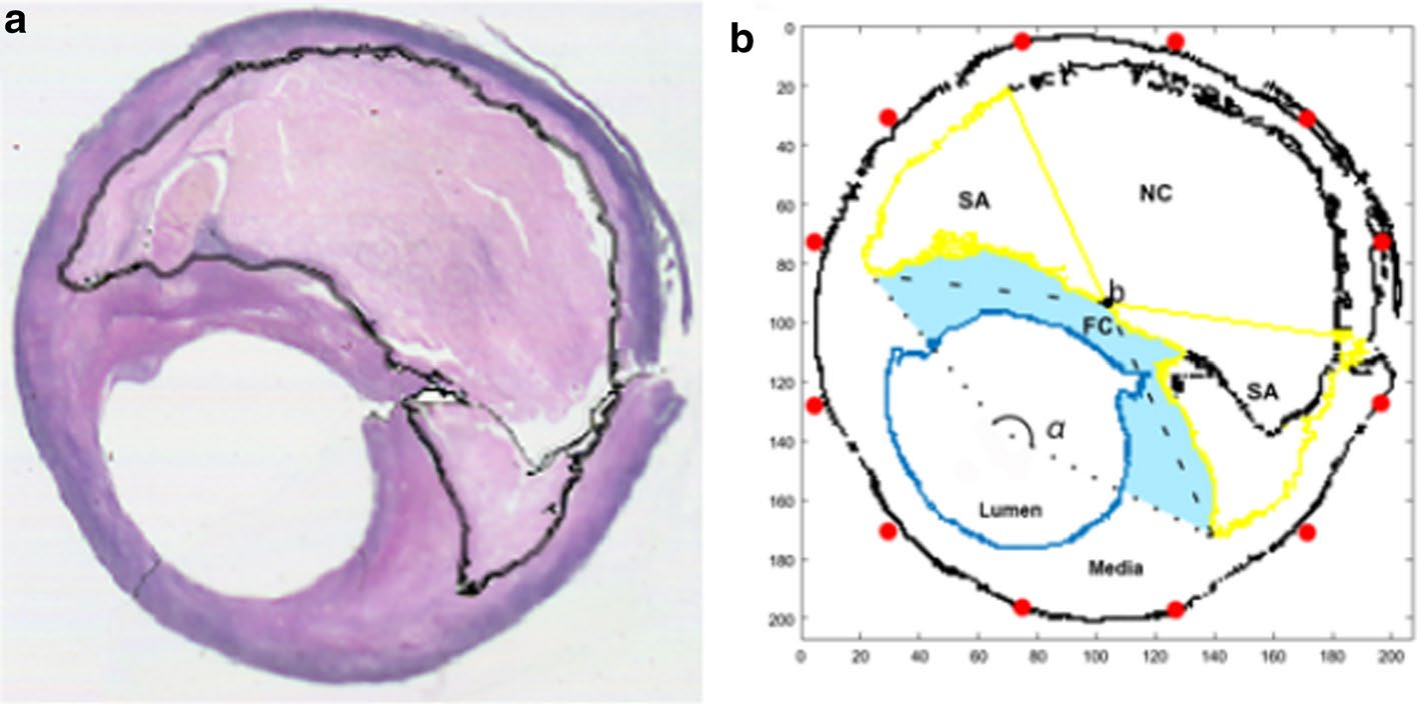
**a** Histology of a patient’s plaque; **b** Schematics of the segmentation of simulation region. The plaque was divided into three different areas: necrotic core (NC), shoulders (SA) and fibrous cap (FC)

A male patient, 76 years old, had an 80% left carotid stenosis. MR imaging study of the carotid plaque was performed using our established protocol [28]. Carotid endarterectomy was performed following the MR imaging study and histological analysis was performed. The histology data with the hematoxylin and eosin (HE) -stained was used to define the region of the model, which is a two-dimensional region of 4 mm $\times$ 4 mm, discreted into 200 $\times$ 200 grids uniformly. We divided the plaque into three different areas: necrotic core (NC), shoulders (SA) and fibrous cap (FC) (Fig. 7) [29]. We first processed the image and outlined the boundary of the plaque area (black line shown in supplemental Fig. 7a). Then we connected the two tips of plaque with the center of the lumen, resulting in an angle alpha (Fig. 7b). The point where the bisector of alpha cut the boundary of plaque was called b. The two tips of plaque were connected with b and their extension lines were used to distinguish the shoulders with the necrotic core. The narrow area between the lumen and the plaque was defined to be the fibrous cap. Twelve sprouts (red dots shown in Fig. 7b) from vasa vasorum were assumed to be located on the media, uniformly with 30 degree steps as the initial condition of intraplaque neovascularization.

By considering four diagonal directions based on the classic two-dimensional five-point (2D5P) modeling of neovascularization proposed by Anderson & Chaplain [30], two-dimensional nine-point (2D9P) model was introduced in our previous published study for solid tumor angiogenesis [22]. In this work, 2D9P model was adopted to describe the neovascularization of intraplaque angiogenic microvessels. The current model focused on six key components involved in the intraplaque angiogenesis, i.e., the ECs density (n), the smooth muscle cells (SMCs) (s), the macrophages (g), VEGF concentration (c), ECM concentration (f), and matrix metalloproteinase (MMPs) concentration (m). The chemical substances were assumed to change with spatial and time, and satisfied the following coupled reaction–diffusion equations:

1 $$\frac{{\partial n}}{{\partial t}} = {D_n}{\nabla ^2}n - \nabla \left( {\frac{{{\chi _0}{k_1}}}{{{k_1} + c}}n\nabla c} \right) - \nabla \left( {{\rho _0}n\nabla f} \right)$$

2 $$\frac{{\partial c}}{{\partial t}} = {D_c}{\nabla ^2}c - \lambda nc + {\eta _c}sc$$

3 $$\frac{\partial f}{\partial t}=-{\eta }_{f}mf$$

4 $$\frac{\partial m}{\partial t}={D}_{m}{\nabla }^{2}m+{\kappa }_{m}n+{\kappa }_{g}g-{\sigma }_{m}m$$

The 2D9P hybrid discrete-continuum angiogenesis model assumed the migration of the ECs through random motility, the chemotaxis in response to the VEGF attraction, and the haptotaxis in response to the ECM adherence Eq. (1). The changes of chemicals satisfied the reaction–diffusion equations (Eqs.(2)–(4)). Specifically, the VEGF was assumed to diffuse, be produced by SMCs and consumed by ECs, with the diffusion coefficient $D_{c}$ and constant parameters $\eta_{c}$ and $\lambda$ respectively. The ECM was degraded by MMPs linearly with coefficient $\eta_{f}$. The MMPs was diffused, be produced by ECs and macrophages, as well as decay by itself, with the diffusion coefficient $D_{m}$ and constant parameters $\kappa_{m} ,\kappa_{g} ,\sigma_{m}$, respectively.

The initial conditions of the three chemicals in Eqs.(2), (3) and (4) were:

5 $$ c(x,y,0) = \frac{{(\nu - r_{1} )^{2} }}{\nu - 0.4771} + \frac{{(\nu - r_{2} )^{2} }}{\nu - 0.4771} $$

6 $$ f(x,y,0) = 1 $$

7 $$ m(x,y,0) = 0 $$

The VEGF was assumed to be a high concentration in the shoulder areas. $\nu$ was a positive constant and $r_{1} ,r_{2}$ were the distances to the center points of the two shoulders (area SA in Fig. 7), respectively.

8 $$ n(x,y,0) = \left\{ {\begin{array}{*{20}c} 1 \\ 0 \\ \end{array} } \right.\begin{array}{*{20}c} {,Sprouts} \\ {,else} \\ \end{array} $$

9 $$ s(x,y,0) = \left\{ {\begin{array}{*{20}c} 0 \\ 1 \\ \end{array} } \right.\begin{array}{*{20}c} {} \\ {} \\ \end{array} \begin{array}{*{20}c} {,\begin{array}{*{20}c} {NC} & {and} & {Lumen} \\ \end{array} } \\ {,else\begin{array}{*{20}c} {} & {} & {} & {} \\ \end{array} } \\ \end{array} $$

10 $$ g(x,y,0) = \left\{ {\begin{array}{*{20}c} 1 \\ 0 \\ \end{array} } \right.\begin{array}{*{20}c} {} \\ {} \\ \end{array} \begin{array}{*{20}c} {,{\text{s}}houlder} \\ {,else\begin{array}{*{20}c} {} & {} \\ \end{array} } \\ \end{array} $$

### Hemodynamic calculation in microcirculation

**Fig. 8:**
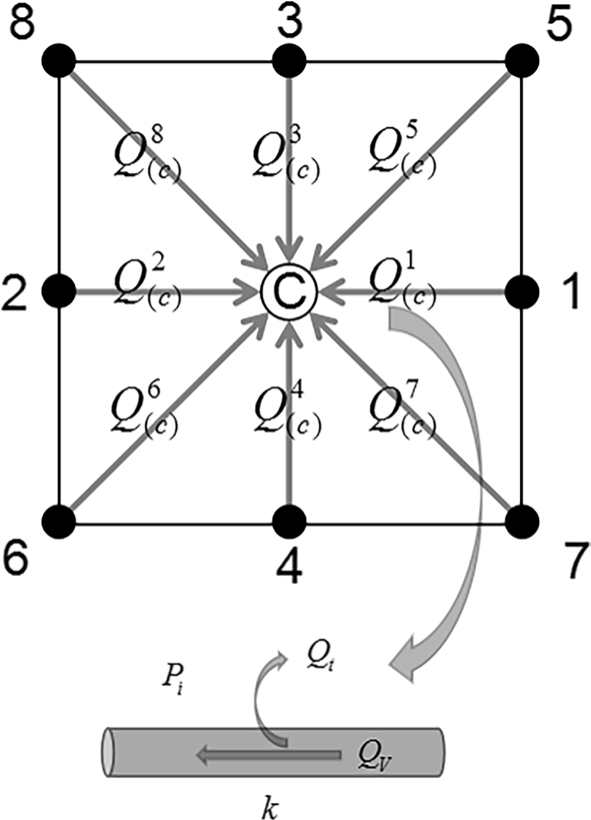
Schematics of the 2D9P angiogenesis model and the blood flow through one segment

There were three parts in the hemodynamic calculation of neovasculatures, i.e., the intravascular blood flow, the interstitial fluid flow, and the transvascular flow (Fig. 8) [25].

Blood flow within point C from its adjacent points (if there was a vessel segment, which represented by a binary matrix $B_{(c)}^{k}$) satisfied the flow conservation equation:

11 $$ \sum\limits_{k = 1}^{8} {Q_{(c)}^{k} B_{(c)}^{k} } = 0{\kern 1pt} {\kern 1pt} $$

12 $$ Q_{(c)}^{k} = Q_{V,(c)}^{k} - Q_{t,(c)}^{k} {\kern 1pt} $$

where $Q_{t,(c)}^{k}$ was the transvascular plasma flow (see Eq. (16)) and $Q_{V,(c)}^{k}$ represented intravascular blood flow without transvascular transport, and followed Poiseuille’s law

13 $$ Q_{V,(c)}^{k} = \frac{{\pi \overline{R}_{k}^{4} }}{{8\overline{\mu }_{k} }}\frac{{(\Delta P_{V,(c)} )}}{{\Delta l_{k} }}{\kern 1pt} $$

The interstitial flow rate $\overrightarrow {{U_{i} }}$ satisfied the continuity equation as well as Darcy’s law [31, 32]:

14 $$ \nabla \cdot \overrightarrow {{U_{i} }} = \sum {Q_{t,(c)}^{k} } $$

15 $$ \overrightarrow {{U_{i} }} = - K\nabla P_{i} {\kern 1pt} {\kern 1pt} $$

where K denoted the hydraulic conductivity coefficient of the interstitial tissue. The transvascular flow $Q_{t,(c)}^{k}$ was governed by Starling’s law:

16 $$ Q_{t,(c)}^{k} = 2\pi \overline{R}_{k} \Delta l_{k} \cdot L_{p} (\overline{P}_{V,(c)}^{k} - \overline{P}_{i,(c)}^{k} - \sigma_{T} (\pi_{V} - \pi_{i} )) $$

$\overline{P}_{V,(c)}^{k}$ and $\overline{P}_{i,(c)}^{k}$ represented the average pressure and the average interstitial pressure in and outside the vessel segment k, respectively. $\sigma_{T}$ was the average osmotic reflection coefficient for plasma; $\pi_{V} ,\pi_{i}$ were the colloid osmotic pressure of blood plasma and interstitial fluid respectively.

S_IPH_ represented the extravascular plasma concentration, which was governed by the extravascular plasma transport throughout the interstitial tissue and the transvascular plasma flow across the leaky wall of the angiogenic microvessels.

17 $$\frac{\partial {S}_{IPH}}{\partial t}=\varphi \cdot {\nabla }^{2}{S}_{IPH}-\Psi {S}_{IPH}\cdot {U}_{i}+\gamma {Q}_{t}$$

To compared the simulation results of IPH with MR images, where $S_{IPH} > S_{IPH}^{Thr}$, we considered it as high IPH regions. $S_{IPH}^{Thr}$ was a threshold determined by the MR images. The baseline parameter values were listed in Table 1.

**Table 1:** The baseline parameter values of the simulation

Parameter	Baseline value	Parameter	Baseline value
*D*_*n*_ (10^−10^cm^2^/s)	1	${\chi }_{0}$(cm^2^/s M)	2600
*k*_*1*_ (10^−10^ M)	7	${\rho }_{0}$(cm^2^/s M)	1000
*D*_*c*_ (10^−7^cm^2^/s)	2.9	$\uplambda$(M^−1^ s^−1^)	750
${\eta }_{c}$(M^−1^$\cdot$s^−1^)	750	${n}_{f}$(cm^3^/s M)	130
*D*_*m*_ (10^−10^cm^2^/s)	1	${\kappa }_{m}$(10^−3^ cm^−3^ s^−1^)	1
${\kappa }_{g}$(10^−3^ cm^−3^ s^−1^)	1	${\sigma }_{m}$(10^−8^ s^−1^)	1
$\upnu$	1.07	*R*_*0*_ (μm)	4 [34]
*K* (10^−9^cm^2^/mmHg·s)	41.3 [33]	*L*_*p*_ (10^−7^ cm/mmHg s)	2.8 [33]
$\sigma_{T}$	0.82 [33]	$\pi_{V}$(mmHg)	20 [33]
$\pi_{i}$(mmHg)	15 [33]	$\phi$	0.4
$\uppsi$	0.05	$\upgamma$	0.4
$\beta$	0.4	*P*_*in*_(mmHg)	10
*P*_*out*_(mmHg)	1		

### Principles of 2D9P angiogenesis model

We used the Euler's nine point finite difference scheme to discretize the above equations [22, 30], in which the discretized equation of ECs was:

18 $$\eqalign{
   n_{i,j}^{q + 1} = n_{i,j}^q{P_0} + n_{i + 1,j}^q{P_1} + n_{i - 1,j}^q{P_2} + n_{i,j + 1}^q{P_3} +\, n_{i,j - 1}^q{P_4}  \cr +\,
   n_{i - 1,j + 1}^q{P_5} + n_{i + 1,j - 1}^q{P_6} + n_{i - 1,j - 1}^q{P_7} + n_{i + 1,j + 1}^q{P_8} \cr} $$

where the subscripts i, j and the superscripts q specified the location on the grid and the time steps, respectively. The 2D9P model assumed that the coefficients P0 ~ P8 were the probability density function to determine the movement direction of the sprouts. The nine coefficients corresponded to the nine possible movements for ECs, including stationary, four horizontal and vertical directions, and four diagonal directions (shown in Fig. 8). In each time step, the probability density functions P0 ~ P8 were integrated into the cumulative distribution function. The probability of the EC to migrate to a certain neighbor grid was set to be proportional to the value of the cumulative distribution function. After the ECs distribution updated, there were two 2D binary matrixes A and B to record the topology of the vessel network. The former was imposed on the simulation grids, while the latter on the vessel segments. The values of both were 1 if the EC/vessel was on the grid/segment, and 0 if it is not.

The new sprouts were assumed to generate only from the existing sprout tips, and the newly formed sprouts were unlikely to branch immediately. Sufficient space locally was requisite for the forming of a new sprout. Furthermore, we assumed that the probability of generating a new sprout was dependent on the local concentration of VEGF. If one sprout tip encountered another sprout or vessels, anastomosis would occur. As a result of the tip-to -tip fusions, only one of the original sprouts continued to grow.

### Simulation algorithms

The simulation algorithms of hemodynamics throughout the neovasculature were as follows:

(a) Set initial pressure P_in_ of 12 entrances and exits where vessels stop growing with pressure P_out_. The initial iteration value is also set.

(b) Solved intravascular pressure $P_{V}$ and its relative error.

In the model, we used iterative method to solve the pressure $P_{V,(c)}$:

19 $$P_{V,(c)}^{m + 1} = \frac{{\sum\limits_{k = 1}^8 {\{ [\frac{{\bar R_k^4}}{{{{\bar \mu }_k}\Delta {l_k}}} - 8{{\bar R}_k}\Delta {l_k}{L_p}]P_{V,(k)}^m}  + 16{{\bar R}_k}\Delta {l_k}{L_p}[\bar P_{i,(c)}^k + {\sigma _T}({\pi _V} - {\pi _i})]\}  \cdot B_{(c)}^k}}{{\sum\limits_{k = 1}^8 ( \frac{{\bar R_k^4}}{{{{\bar \mu }_k}\Delta {l_k}}} + 8{{\bar R}_k}\Delta {l_k}{L_p}) \cdot B_{(c)}^k}}$$

20 $$ errP_{V} = \sum {\left| {P_{V,(j,k)}^{{}} - P_{V,(j,k)}^{o} } \right|} /\sum {P_{V,(j,k)} } {\kern 1pt} {\kern 1pt} $$

(c) Calculated interstitial pressure *P*_*i*_ and its relative error.

Using FTCS discrete equation of (14), (15) and (16):

21 $$\frac{{P_{i,(j + 1,k)}^{m} - 2P_{i,(j,k)}^{m + 1} + P_{i,(j - 1,k)}^{m} }}{{\Delta x^{2} }} + \frac{{P_{i,(j,k + 1)}^{m} - 2P_{i,(j,k)}^{m + 1} + P_{i,(j,k - 1)}^{m} }}{{\Delta y^{2} }} = \alpha^{2} (P_{i,(j,k)}^{m + 1} - P_{e,(j,k)} )A_{j,k}.$$

In the model, the successive over-relaxation (SOR) iteration method was used to solve the formula,$\Omega$ was the coefficient of relaxation:

22 $$ \begin{gathered} P_{i,(j,k)}^{m + 1} = P_{i,(j,k)}^{m} + \frac{\omega }{{4 + \Delta x^{2} \alpha^{2} A_{j,k} }}[\Delta x^{2} \alpha^{2} A_{j,k} P_{e,(j,k)} + P_{i,(j + 1,k)}^{m} \\ + \,P_{i,(j - 1,k)}^{m} + P_{i,(j,k + 1)}^{m} + P_{i,(j,k - 1)}^{m} - (4 + \Delta x^{2} \alpha^{2} A_{j,k} )P_{i,(j,k)}^{m} ]{\kern 1pt} {\kern 1pt} {\kern 1pt} \\ \end{gathered} $$

23 $$ errP_{i} = \sum {\left| {P_{i,(j,k)} - P_{i,(j,k)}^{o} } \right|} /\sum {P_{i,(j,k)} } $$

where Pe was the effective pressure of capillary and equaled to $P_{e} = P_{V} - \sigma_{T} ({\pi_{V}} - {\pi_{i}} )$(d) Estimated the maximal iterative error, repeat b) to c) until $errP_{V} \le 10^{ - 6} {\kern 1pt} {\kern 1pt} {\kern 1pt} and{\kern 1pt} {\kern 1pt} {\kern 1pt} errP_{i} \le 10^{ - 6}$.

(e) Calculated the intravascular flow Qv, and the transvascular flow $Q_{t}$ using Eqs. (13 and 16).

(f) Calculated the interstitial fluid velocity Ui using Eqs.(14) and (15).

(g) Calculated intraplaque hemorrhage area matrix $S_{IPH}$ (Eq. 17).

## Data Availability

The datasets used and/or analysed during the current study are available from the corresponding author on reasonable request.
